# Estimation of the Cellular Antioxidant Response to Chromium Action Using ESR Method

**DOI:** 10.1100/tsw.2004.136

**Published:** 2004-09-02

**Authors:** Tamar Kartvelishvili, Marina Abuladze, Nino Asatiani, Joseph Akhvlediani, Eugene Kiziria, Lali Asanishvili, Lia Lejava, Hoi-Ying N. Holman, Nelly Sapojnikova

**Affiliations:** ^1^ Institute of Molecular Biology and Biophysics, Georgian Academy of Sciences, 14, Gotua Str., 0160 Tbilisi, Georgia; ^2^ Institute of Physics, Georgian Academy of Sciences, 6, Tamarashvili Str., 0177 Tbilisi, Georgia; ^3^ Center for Environmental Biotechnology, E.O. Lawrence Berkeley National Laboratory, 1, Cyclotron Road, Berkeley, CA, 94720, USA

**Keywords:** chromium (VI), antioxidant systems, cell culture, ESR

## Abstract

In the present study, the antioxidant capacity of chromium-treated L-41 (human epithelial-like cells) was investigated by the ESR spin-trapping technique. The crude cell extracts of the cells grown in the presence of 2 µM (nontoxic) and 20 µM (toxic) chromium (VI) concentrations were tested in the model Fenton system with and without catalase-inhibitor sodium azide. The presented approach using the ESR technique along with inhibitors lets us discern cell extract defense capacity connected with the enzymatic activity in viable cells and the catabolic activity in dying cells.

### INTRODUCTION

Chromium is a widespread industrial chemical that is known to cause toxic and carcinogenic effects in humans and animals[1,2,3,4]. Chromium (Cr) exists primarily in two valence forms: Cr(VI) and Cr(III). The chromate ion $[CrO_4]^{2-}$, the dominant form of Cr(VI) in neutral aqueous solutions (at physiological conditions), crosses cellular membranes via the nonspecific anion transport system ($SO_4^{2-}$ and $HPO_4^{2-}$ channels) and is biologically active[5,6,7,8]. Cr(VI) alone does not react with isolated DNA *in vitro*[9]. According to the uptake-reduction model of Cr(VI) carcinogenicity[5,10], within a cell, Cr(VI) undergoes reduction to lower oxidation states Cr(V/IV/III) by different intracellular reductants, such as glutathione (GSH), cystein, ascorbic acid, and glutathione reductase (GR)[11,12,13,14]. In contrast to Cr(VI), Cr(III) does not easily penetrate cell membranes, but once inside cells, Cr(III) (as the final product of Cr[VI] reduction) can produce Cr(III)-DNA adducts[15].

As a transient metal, Cr(VI) and its reduced forms can form the coordinated complexes with the mentioned reductants[16] that cause (*in vitro* and *in vivo*) oxidative modification of the metal-complexing ligands and a wide variety of DNA lesions, such as Cr-DNA adducts, DNA strand breaks, and DNA-DNA cross-links[17,18,19,20,21,22].

During Cr(VI) reduction, a wide variety of reactive intermediates, such as oxygen-, carbon-, and sulfur-centered radicals formed from complexing ligands[16], may initiate a series of radical reactions that can have serious pathological consequences. Besides these intermediates, elevated levels of many forms of reactive oxygen species (ROS) generated under Cr(VI) action, such as superoxide anion ($O_2^{\cdot -}$), hydrogen peroxide ($H_2O_2$), singlet oxygen ($O_2^1$), and ·OH radicals (which are the products of normal cellular metabolism as well), can be actually involved in the processes leading to the oxidative stress under chromium action.

The general proposed mechanism of oxygen activation by Cr(VI) is provided by Fenton–like and Haber-Weiss reactions including Cr(VI/V), Cr(V/IV), Cr(IV/III), and Cr(III/II) redox pairs[23,24]. Substrates of mentioned reactions are $O_2^{\cdot -}$ and $H_2O_2$, which realize redox cycling of chromium, resulting in Haber-Weiss reaction ($O_2^{\cdot -} + H_2O_2 \rightarrow O_2 + \cdot OH + OH^-$)[25].

In cells, the toxic capacity of Cr(VI) can be decreased by the defense system. The cellular antioxidant system consists of nonenzymatic (α-tocopherol, ascorbic acid, GSH) and enzymatic (catalase, superoxide dismutases [SODs], glutathione peroxidase [GPx], GR) antioxidants. When the defense system of the cell is overwhelmed and redox homeostasis is altered, the result is an oxidative stress[26].

In the present study, we have examined the antioxidant capacity of chromium-treated cells in the presence of $H_2O_2$, which especially increases in some pathological conditions. The study was conducted by the electron spin resonance (ESR) spin-trapping technique. This technique was already used in our previous study to compare the antioxidant capacity against $H_2O_2$ in the two distinct cell lines, L-41 (human epithelial-like cells) and HLF (human diploid lung fibroblasts), with different antioxidant enzyme activity[27]. The antioxidant capacity of L-41 cells treated with Cr(VI) has been compared with the activities of particular antioxidants in response to Cr(VI) action. The presented approach enables us to estimate the total cell antioxidant capacity without the separate measurement of the activities of the cell antioxidants.

### METHODS

#### Cell Culture

The L-41 cell line is a human epithelial-like cell line that was derived from the J-96 cell line originally obtained from a patient with monocytic cell leukemia (Research Center of Medical Genetics, Russian Academy of Medical Science, Moscow)[28,29]. The L-41 cells were maintained as adherent cells in Eagle's culture medium supplemented with 10% donor calf serum, 2 mM L-glutamine, 100 units of penicillin/ml, and 100 μg of streptomycin/ml at 37ºC in a 5% $CO_2$ incubator. Cells were harvested with trypsin (0.25%)/EDTA solution. L-41 cells represent an immortalized cell line with stable phenotype in cell culture and high proliferate activity.

#### Glutathione Antioxidant System

Glutathione concentration was determined by using GSH-400 colorimetric assay (Oxis, USA). Glutathione reductase (GR) activity was measured by using the BIOXYTECH GR-340™Assay (Oxis, USA). Glutathione peroxidase (GPx) activity was determined by using BIOXYTECH GPx-340™ colorimetric assay for cellular GPx (Oxis, USA).

### Catalase Activity

Catalase at different time points and chromium concentrations, and without chromium action as well, was detected in the crude cell extracts. Catalase activity was determined by measuring the rate of $H_2O_2$ (10 mM) decomposition in 50 mM potassium phosphate buffer, (pH 7.0), in the presence of the crude cell extract at 240 nm and 25°C, $\varepsilon_{H2O2} = 43.6\ M^{-1}\ cm^{-1}$[30].

### Preparation of the Crude Cell Extract

Cr(VI) as potassium chromate (2 and 20 μM) was added to the cell culture at 48 h of growth (80% of confluence) and the cells continued to grow under permanent Cr(VI) action for different time periods (24 and 48 h). Cells (~$10^7$) grown without Cr(VI) or under Cr(VI) action were harvested by centrifugation (3,000 rpm, 5 min, 4ºC), rinsed twice in 50 mM phosphate buffer, pH 7.8. The rinsed cells were resuspended in a definite volume of above-mentioned buffer 1:4 (w/v), sonicated five times for 10-sec bursts (44 kHz), centrifuged (14,000 rpm, 20 min, 4ºC), and the soluble extract was used as a sample. The crude cell extracts were standardized per microgram of total protein. Protein concentrations in the cell extract were determined using BCA (bicinchoninic acid) protein assay reagent (Pierce, USA).

### ESR Measurements

Fenton reaction, a well-known ·OH generator, has been chosen as the artificial model system of ROS generation: $Fe^{2+} + H_2O_2 \rightarrow Fe^{3+} + OH^- + \cdot OH$. $H_2O_2$ reacts with free ferrous iron to form toxic and highly reactive hydroxyl radicals.

The ESR spin-trapping technique was used to detect short-lived ·OH radicals in model Fenton reaction. ·OH radicals were trapped by 5,5-dimethyl-1-pyrroline N-oxide (DMPO, Sigma). ESR spectra were obtained by using an ESR spectrometer RE 1306 (Russia) at 100-kHz modulator frequency, 1.2-G modulation amplitude, 25-mM microwave power, microwave frequency 9.3 GHz. The samples were placed in the ESR cavity using cell-glass capillaries with an internal diameter of 1 mm. Spectra were recorded at ambient temperature. The ESR spectrum of DMPO/·OH adducts consists of 1:2:2:1 quartet with splitting of $a_H = a_N = 14.8$ G, where $a_N$ and $a_H$ denote hyperfine splitting of the nitroxyl nitrogen and β-hydrogen, respectively, which is typical of this system[31]. The ratio of the second signal intensity of DMPO/·OH adduct to the intensity of the reference (DPPH-1,1-diphenyl-2-picryl-hydrazyl as a reference) was estimated and is presented as arbitrary units (A.U.) in Figs. 1 and 2. The reaction mixture contained 100 mM DMPO, 1 mM $FeSO_4$, 100 mM $H_2O_2$, and 50 mM sodium-potassium buffer pH 7.4 in a final volume 62.5 μl. The antioxidant capacity of the cells was registered for the definite periods of time after the addition of the cell crude extracts to the model Fenton reaction. Protein concentration in ESR sample was 0.168 mg/ml.

## RESULTS AND DISCUSSION

### Estimation of the Antioxidant Defense System Activity

ROS taking part in Fenton (or Fenton-like) and Haber-Weiss reactions can be extensively neutralized by complex and carefully balanced antioxidant enzymes, of which the SODs are the initial enzymes converting superoxide anion to $H_2O_2$, one of the stress-inducing agents potentially capable of forming oxidative intermediates in cells. $H_2O_2$ can be decomposed primarily by catalase ($H_2O_2 \rightarrow H_2O + O_2$) (in peroxisoms) and by GPx ($H_2O_2 \rightarrow H_2O$) (in the cytoplasm and mitochondria) via GSH dependent cycle, which is a mechanism in scavenging alkylhydroperoxides and is a complementary to catalase in scavenging[32].

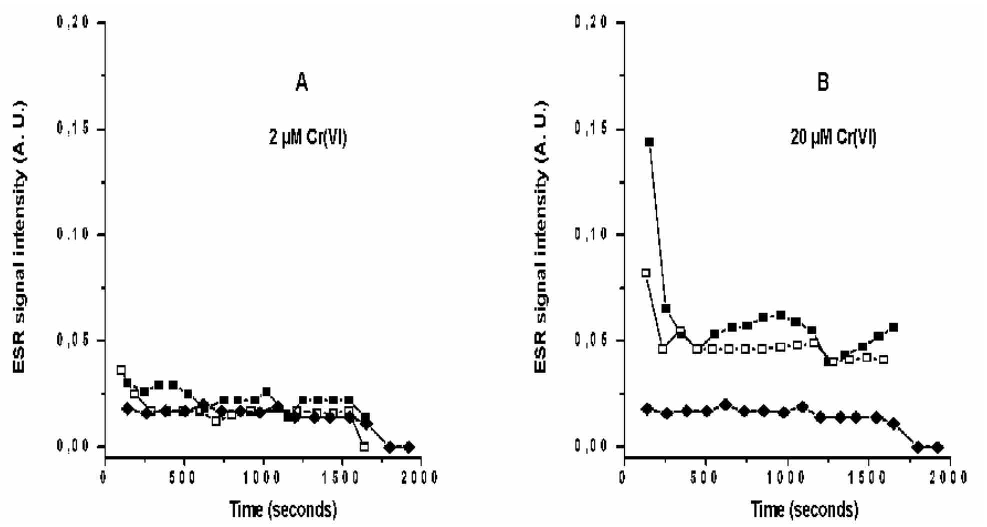

**FIGURE 1**. The time course of the ESR signal intensity in the model Fenton reaction in the presence of crude cell extracts of Cr(VI)-treated and -untreated control L-41 cells. (A) 2 µM Cr(VI) treatment; (B) 20 µM Cr(VI) treatment. ◆, Control; □, 24 h of the cell growth under Cr(VI) action; ■, 48 h of the cell growth under Cr(VI) action. Cr(VI) as potassium chromate was added to the cell culture at 48 h of growth (80% of confluence). The model Fenton reaction mixture contained 100 mM DMPO, 1 mM $FeSO_4$, 100 mM $H_2O_2$, and 50 mM sodium/potassium buffer pH 7.4 in a final volume 62.5 µl. Protein concentration in ESR sample was 0.168 mg/ml.

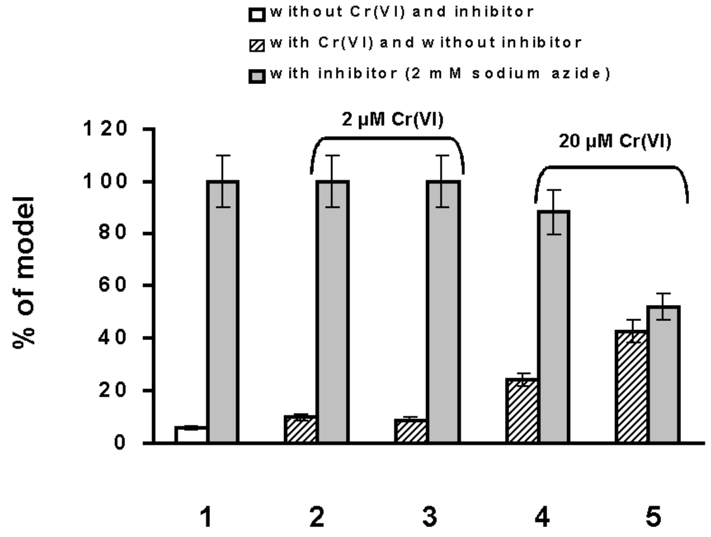

**FIGURE 2**. Effects of sodium azide inhibitory action on the antioxidant capacity of the crude cell extracts of L-41 cells estimated by ESR method. (1) Cells grown without Cr(VI) treatment; (2 and 4) after 24 h of Cr(VI) treatment; (3 and 5) after 48 h of Cr(VI) treatment. Cr(VI) as potassium chromate was added to the cell culture at 48 h of growth (80% of confluence). The experimental conditions are the same as described in Fig. 1. The ESR signal intensity of the model Fenton reaction is assigned as 100%. The data presented are mean values ± S.D. from three separate sets of experiments.

In our previous study, we characterized the effect of long-term Cr(VI) action in the ranges of 2–20 µM on cultured human cells L-41. The time-course action of 2 µM of Cr(VI) demonstrated that this concentration did not affect cell viability up to 96 h. The cell treatment with 20 μM of Cr(VI) increased apoptotic cells fraction determined by morphological changes which corresponded with apoptosis up to 20–30% of cell number at 24 h, and up to 80–90% at 48 h of Cr(VI) action. Two other sensitive markers, namely, genomic DNA fragmentation and caspase-3 activation, demonstrated induction of the apoptotic cell death. Thus, the time-dependent loss of cell viability at the cell exposure to 20 μM of Cr(VI) was accompanied by the increased level of apoptotic cell[33].

The changes of the antioxidant defense system in response to Cr(VI) action are presented in Table 1. The action of 20 μM of Cr(VI) for 48 h resulted in the complete exhaustion of the GSH cycle system, whereas catalase remained at the rather high level. Hence, the major defense against $H_2O_2$ still assists in cells.

**TABLE 1**
**Defense System Activity of L-41 Cells in Response to Cr(VI) Action**

| Control (time of growth, h)/Cr(VI) μM (time after Cr(VI) addition, h) | Catalase (U/ml) | GPx (mU/ml) | GR (mU/ml) | GSH (μM) |
|---|---|---|---|---|
| Control (72 h) | 180 ± 27 | 133 ± 20 | 101 ± 17 | 450 ± 43 |
| 2 μM Cr(VI) (24 h) | 310 ± 46 | 206 ± 31 | 76 ± 11 | 170 ± 26 |
| 20 μM Cr(VI) (24 h) | 120 ± 18 | 51 ± 8 | 20 ± 4 | 420 ± 56 |
| Control (96 h) | 300 ± 37 | 66 ± 10 | 70 ± 12 | 600 ± 55 |
| 2 μM Cr(VI) (48 h) | 280 ± 25 | 41 ± 5 | 87 ± 9 | 750 ± 60 |
| 20 μM Cr(VI) (48 h) | 130 ± 20 | N.D. | N.D. | N.D. |

Each value is the mean ± S.D. from three separate sets of experiments. Significant difference in the enzyme activities and glutathione quantity ($p < 0.05$, Student's t-test) was obtained between the control untreated and Cr(VI)-treated cells. Cr(VI) was added to the cell culture at the 48 h of growth and the cells continued to grow under permanent Cr(VI) action for 24 and 48 h. N.D. – nondetected (under the threshold of detection).

The cell exposure to 2 μM of Cr(VI) for 48 h did not cause depletion of the antioxidant defense system (GSH, GPx, GR, catalase), which was in agreement with the lack of the cytotoxicity.

### Estimation of the Cell Antioxidant Capacity by ESR

The intracellular antioxidant capacity of cells was estimated by the ESR spin-trapping technique in an artificial Fenton reaction system, a common source of ·OH radicals, which are formed via $Fe^{+2}$ ion interaction with $H_2O_2$. Hydroxyl radicals were trapped by DMPO.

The antioxidant capacity of cells was registered for the definite periods of time after addition of the crude cell extracts to the model Fenton reaction. As catalase and GPx (acting via its cofactor GSH) are main scavengers of $H_2O_2$, addition of the crude cell extract to the model Fenton reaction resulted in the decrease of ·OH radical level. The crude cell extracts induced the suppression of DMPO/·OH spin-adduct signal from the outset. The suppression capacity was examined in time-dependent manner.

As it follows from Fig. 1A, cells exposed to 2 μM of Cr(VI) reveal the high suppressing capacity against formation of the hydroxyl radical, similar to the control untreated cells. The time-course of the ESR signal intensity in model Fenton reaction in the presence of the crude cell extracts of L-41 cells grown without chromium for 72 and 96 h (controls) did not differ (data not shown). These data agree with the lack of cytotoxicity at the 2 μM of Cr(VI) action for 48 h and should be attributed to the elevated activity of some antioxidants in response to chromium action after 24 h and to the restoration of antioxidant activity to the control level in the next 24 h (Table 1).

The intracellular antioxidant capacity of chromium-induced apoptotic L-41 cells deteriorated as compared with the control cells, but it was still high (Fig. 1B). We were surprised by the high defensive capacity of L-41 cells grown under toxic 20 μM of Cr(VI) action for 48 h. As mentioned above, at this time point, the basic enzymes (such as GPx, GR, and low molecular weight antioxidant GSH) are completely depleted, and they cannot defense cells. Catalase activity is decreased compared to control (96 h of the cell growth), but it is not depleted. Hence the defense could be accomplished by catalase and/or by products of the cell catabolism, which could be presented at high level in apoptotic cells as the result of the activation of catabolic enzymes.

The catabolic processes providing the utilization of protein and nucleotide excess play the key role at the late stages of oxidative stress, when the depletion of the cell energy sources takes place. Mononucleotides, obtained at the decomposition of nucleic acids, and proteins are generally hydrolyzed forming low molecular weight nitrogen-containing compounds (amino acids, peptides, urea, uric acid, etc). These products along with the other low molecular weight compounds (glucose, thiourea, uracyl, and alcohol) promote the inhibition of ·OH radicals, and hence these products could accomplish the antioxidant defense at apoptosis.

### Estimation of the Inhibitor Action

Generally, catalase and GPx are thought to be the most specific enzymes for $H_2O_2$ decomposition, although other enzymes such as peroxidases and peroxiredoxins have $H_2O_2$ decomposition capacity.

To estimate the unexpected high suppression capacity of L-41 cells grown under permanent exposure to 20 μM of Cr(VI) and to confirm the possible role of catabolic products in the intracellular antioxidant capacity of apoptotic cells, the effect of a catalase inhibitor, such as sodium azide ($NaN_3$), was investigated. It was reported that in the presence of $H_2O_2$, catalase was reduced by the addition of $NaN_3$. $NaN_3$ is an inhibitor responsible for the inactivation of heme enzymes including catalase by directly attaching to the coordination position of the iron in the heme moiety and producing an inactive ferrous derivative[34,35].

In all discussed cases, 2, 4, and 6 mM $NaN_3$ added to the crude cell extracts caused the same effect (data not shown). Hence, 2 mM of $NaN_3$ is completely capable of inhibiting catalase existing in the crude cell extracts. $NaN_3$ added to the crude cell extracts provoked the increase of ·OH radical levels via inhibition of catalase, but to different extent. In the case of control cells (grown without Cr[VI]) and cells exposure to 2 μM of Cr(VI), the ·OH radical level achieved the model system level (Fig. 2). After the inhibition of the catalase activity by $NaN_3$, the level of ·OH radical was not to reach the model level, as other uninhibited enzymes responsible for $H_2O_2$ decomposition could remain in the cell extract. Such a high level of ·OH radicals could be connected with a high contribution of catalase compared with other enzymes decomposing $H_2O_2$ and/or SOD pro-oxidant effect. It was observed that SOD could provoke a toxic effect[36]. This effect is bound to the peroxidatic activity of Cu,Zn-SOD (nonspecific function) causing ·OH radical production via Fenton-like reaction[23,27,37].

At the cell exposure to 20 μM of Cr(VI), catalase activity decreased (Table 1), but it was still high and remained about at the same level after 24 and 48 h of Cr(VI) action. In spite of this, $NaN_3$ action induced different effects on the antioxidant capacity of the crude cell extracts. After 24 and 48 h of 20 μM of Cr(VI), permanent action ·OH radical levels achieve 88 and 55% of the model Fenton system, respectively. As 2 mM of $NaN_3$ was sufficient for total inhibition of catalase, catalase is probably not completely responsible for the defensive capacity against $H_2O_2$ at 48 h of toxic chromium action. The influence of the SOD pro-oxidant effect on the ·OH radical level is not likely to be true, as Cu,Zn SOD was practically exhausted in 48 h of 20 μM of Cr(VI) action[33].

The activation of caspase-3 at 24 h of Cr(VI) action leads to the formation of the elevated intracellular level of amino acids and short oligopeptides as the result of cleavage of a variety of intracellular polypeptides. Another intracellular change typical of apoptosis is the degradation of the chromosomal DNA into high molecular weight and oligonucleosomal fragments. The remarkable DNA ladder (oligonucleosomal fragmentation) was observed after 48 h of exposure of L-41 cells to 20 μM of Cr(VI)[33]. Thus, apoptosis involves activation of catabolic mediators and enzymes[38]. Apoptosis and especially a late stage of the apoptotic process generally trigger production of above-mentioned catabolic compounds, which possess scavenging capacity against hydroxyl radical. Probably, it is a reason of the high suppressing capacity of L-41 cells exposed to 20 μM of Cr(VI) in the model Fenton reaction.

The presented approach using the ESR technique along with inhibitors lets us discern the cell extract defense capacity connected with the enzymatic activity in viable cells and the catabolic activity in dying cells. The use of different inhibitors in the proposed approach could raise the possibility of distinguishing the contribution of nonenzymatic and enzymatic antioxidants to the defense against various stress factors in viable cells.

### ACKNOWLEDGMENT

The authors would like to thank Dr. M. Iobadze (Institute of Medical Biotechnology, Georgian Academy of Sciences, Tbilisi) for her kind gift of the L-41 cell line used in this study. This study was supported by G-349 Grant awarded by the International Science and Technology Center (ISTC).

### BIOSKETCHES

**Tamar Kartvelishvili, PhD,** is a senior researcher in the Department of Peptide Synthesis at the Institute of Molecular Biology and Biophysics (Tbilisi, Georgia). Dr. Kartvelishvili received a B.S. (in organic chemistry) from the Tbilisi State University in 1974 and a PhD (in polymer chemistry) from the Institute of Organoelement Compounds (Moscow, Russia) in 1986. Her research interests focus on synthesis and study of new heterochain biodegradable polymers composed on naturally occurring amino acids and other nontoxic building blocks, and investigation of mechanisms of intracellular chromium reduction and investigation of the cell metabolism pathways in response to heavy metal actions. She was awarded by ISF (G. Soros Foundation, 1994–1995) grant, by JSPS (Kyoto University, 1995) grant, by Research grant at Cornell University, USA (2000), by ISTC grants G-348 (2000–2002) and G-349 (2001–2003). Dr. Kartvelishvili is an author of 42 articles.

**Marina Abuladze, PhD**, was a predoctoral fellow in the Laboratory of Cytogenecity and Cell Morphology at the Institute of Molecular Biology (Moscow, Russia). She received her doctorate in cytology from the Biological Department of Moscow State University in 1976. Currently, she is a senior researcher at the Andronikashvili Institute of Physics (Tbilisi, Georgia). Her work is centered on cell biology, particularly on cell proliferation and growth regulation. At present, investigation of mechanisms of intracellular chromium reduction and investigation of the cell metabolism pathways in response to heavy metal actions is the main field of her investigation. She was awarded by ISTC grants G-348 (2000–2002) and G-349 (2001–2003). Dr. Abuladze is an author of more than 20 articles for biochemical and cell research journals.

**Nino Asatiani, PhD**, was a predoctoral fellow in the Laboratory of Protein Research at the Institute of Molecular Biology (Moscow, Russia). She received her doctorate in biochemistry from the Institute of Plant Biochemistry (Tbilisi, Georgia) in 1985. Currently, she is a senior researcher at the Andronikashvili Institute of Physics (Tbilisi, Georgia). Her research efforts focus on the biochemistry of chromatin, specifically the histone modification. At present, investigation of mechanisms of intracellular chromium reduction and investigation of the cell metabolism pathways in response to heavy metal actions is the main field of her investigation. She was awarded by ISTC grants G-348 (2000–2002) and G-349 (2001–2003). Dr. Asatiani is an author of more than 20 articles for biochemical, biophysical, and molecular biology journals.

**Joseph Akhvlediani, PhD**, is a senior researcher in the Department of NMR at the Andronikashvili Institute of Physics (Tbilisi, Georgia). Dr. Akhvlediani received a B.S. (Physics) from the Tbilisi State University in 1961. He was a predoctoral fellow in the Karpov Physical Chemistry Institute (Moscow, Russia). He received a PhD from the Kazan State University (Russia) in 1969. His research interest is focused on radiation physics of the solid body, superconductivity, and ESR investigation of biological systems (cells and tissues). He is awarded by ISTC grants. Dr. Akhvlediani is an author of 38 articles.

**Eugene Kiziria, PhD**, is a Leading Scientific Researcher in the Department of Physics of Biological Systems at the Andronikashvili Institute of Physics (Tbilisi, Georgia). He received his PhD in biophysics (1975) from Tbilisi State University. He studied kinetics of collagen melting. His recent interests lie in the field of physics of biopolymers and he is also skilled in scientific instrumentation (30 publications). He was awarded from ISTC Project G-349 (2001–2003), G-408 (2001–2004).

**Lali Asanishvili** received a B.S. from Tbilisi State University (Biology) in 1976. Currently, she is a science researcher at the Andronikashvili Institute of Physics (Tbilisi, Georgia).

**Lia Lejava** received a B.S. from Tbilisi State University in physics in 1975. Before starting graduate work, she served as the laboratory assistant at the Andronikashvili Institute of Physics (Tbilisi, Georgia). Currently she is a science researcher at the same institute.

**Hoi-Ying N. Holman** is a principal investigator at the Lawrence Berkeley National Laboratory, Berkeley, California. She received a PhD in the School of Engineering, University of California at Berkeley.

**Nelly Sapojnikova, PhD**, was a predoctoral fellow in the Laboratory of Chromatin Research at the Institute of Cytology (St. Petersburg, Russia). She received her doctorate in biophysics from the Andronikashvili Institute of Physics (Tbilisi, Georgia) in 1989. Currently, she is a senior researcher at the Andronikashvili Institute of Physics. Her research efforts focus on the molecular biology of chromatin. At present, investigation of mechanisms of intracellular chromium reduction and investigation of the cell metabolism pathways in response to heavy metal actions is the main field of her investigation. She was awarded by INTAS grant (1995–1996), by Wellcome Trust grant (1998–2001), by the grant from Austrian Academy of Science (1999), by the grants from ISTC (G-348, G-349). She is an author of 23 articles for biochemical, biophysical, and molecular biology journals.
